# Computed tomographic imaging characteristics of the normal canine lacrimal glands

**DOI:** 10.1186/1746-6148-10-116

**Published:** 2014-05-22

**Authors:** Allison L Zwingenberger, Shin A Park, Christopher J Murphy

**Affiliations:** 1 Department of Surgical and Radiological Sciences, School of Veterinary Medicine, Davis, CA 95616, USA; 2 Department of Ophthalmology & Vision Science, School of Medicine, University of California, Davis, Davis, CA 95616, USA

**Keywords:** Lacrimal gland, Accessory lacrimal gland of third eyelid, CT, Canine

## Abstract

**Background:**

The canine lacrimal gland (LG) and accessory lacrimal gland of the third eyelid (TEG) are responsible for production of the aqueous portion of the precorneal tear film. Immune-mediated, toxic, neoplastic, or infectious processes can affect the glands directly or can involve adjacent tissues, with secondary gland involvement. Disease affecting these glands can cause keratoconjunctivitis sicca, corneal ulcers, and loss of vision. Due to their location in the orbit, these small structures are difficult to evaluate and measure, making cross-sectional imaging an important diagnostic tool. The detailed cross-sectional imaging appearance of the LG and TEG in dogs using computed tomography (CT) has not been reported to date.

**Results:**

Forty-two dogs were imaged, and the length, width, and height were measured and the volume calculated for the LGs & TEGs. The glands were best visualized in contrast-enhanced CT images. The mean volume of the LG was 0.14 cm^3^ and the TEG was 0.1 cm^3^. The mean height, width, and length of the LG were, 9.36 mm, 4.29 mm, and 9.35 mm, respectively; the corresponding values for the TEG was 2.02 mm, 9.34 mm, and 7.90 mm. LG and TEG volume were positively correlated with body weight (p < 0.05).

**Conclusions:**

Contrast-enhanced CT is a valuable tool for noninvasive assessment of canine lacrimal glands.

## Background

The lacrimal gland (LG) is an epithelial gland that is responsible for approximately 60% of the production of the aqueous component of the precorneal tear film in the canine eye, with the remaining 40% contributed by the accessory lacrimal gland of the third eyelid (TEG) [1]. The lacrimal gland can be involved in immune-mediated, toxic, infectious, or cancerous processes [2-4]. Compromise of the LG and TEG can lead to the development of keratoconjunctivitis sicca (KCS) [5] with vision debilitating sequelae [6]. Imaging techniques, such as ultrasound and MR, have become important in detecting and monitoring disease in people and in distinguishing lacrimal gland origin disease from other disorders of the orbit [7-12]. Additional potential uses of advanced imaging include measuring response to therapy and targeted imaging with labeled therapeutics.

The LG is an oval gland that conforms to the dorsolateral surface of the globe. The gland is located deep to the orbital ligament [13]. The LG has been described in humans, rabbits, cattle, and camels as bilobed [8,12,14-17]; other mammals, including the dog, have a single-lobed gland [18,19]. In dogs, the lacrimal gland is ventral to the M. rectus dorsalis and M. levator palpebrae superioris, superficial to the M. rectus lateralis, and dorsal to the M. rectus ventralis [18]. The TEG surrounds the base of the vertical cartilage of the third eyelid [13]. The contents of the orbit and the location of the lacrimal gland have been described on CT images [20,21], however to the authors’ knowledge, the detailed cross-sectional imaging characteristics of the canine lacrimal glands have not been studied.

The purpose of this study was to describe the computed tomographic (CT) imaging characteristics, dimensions, and volume of the normal canine LG and TEG.

## Results

Forty-two dogs were identified with CT imaging studies of the skull, which included the lacrimal glands. Breeds included Australian Cattle Dog (1), Australian Shepherd (2), Border Collie (2), Boxer (1), Chihuahua (2), Cocker Spaniel (1), Doberman Pinscher (2), English Pointer (1), Fox Terrier (2), German Shepherd Dog (1), German Shorthaired Pointer (1), Golden Retriever (3), Labrador Retriever (3), Old English Sheepdog (1), Pit Bull Terrier (2), Pomeranian (1), Rhodesian Ridgeback (1), Rottweiler (3), Samoyed (1), Schnauzer (1), Siberian Husky (1), Soft Coated Wheaten Terrier (1), Standard Poodle (1), Welsh Corgi (1), and mixed breed (6). The mean age of dogs was 8.4 y (SD 3 y, range 1–13 y), and the mean body weight was 28 kg (SD 13 kg, range 4.8-64 kg). Males and females were equally represented (17FS/1 F/21MC/3 M).

Forty-one of 42 dogs in the CT group had visible TEGs, and all 42 had visible LGs. The lacrimal glands (LG and TEG) were not well visualized on unenhanced CT images, and precontrast imaging attenuation was determined by matching images and regions of interest with the postcontrast images.

**Figure 1:**
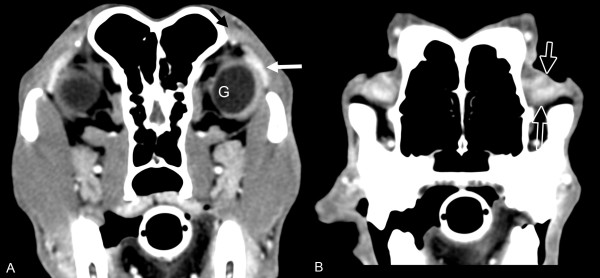
**Transverse contrast-enhanced CT images of the lacrimal glands.** Image markers **A-B** denote the right side of the image. **(A)** The lacrimal gland (white arrow) and **(B)** accessory lacrimal gland of the third eyelid (open arrows) are markedly contrast-enhancing structures on postcontrast 1.25 mm CT images.

**Figure 2:**
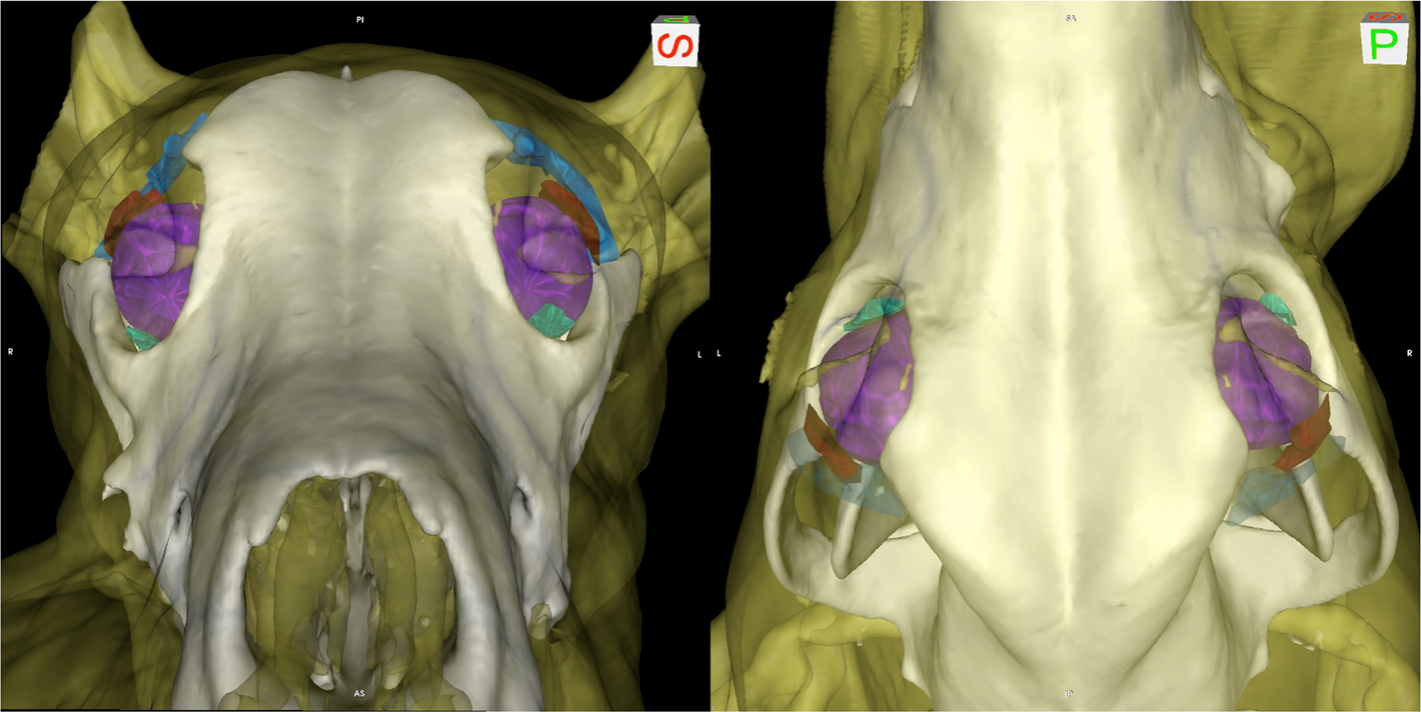
**3D surface rendering of 1.25 mm postcontrast CT images demonstrating the relationship between the globe, the orbital ligament, the accessory lacrimal glands of the third eyelid (green), and the lacrimal glands (red). (A)** View from rostral to caudal with right on the left side of the image, **(B)** view from dorsal with rostral at the top of the image.

On CT images, the lacrimal glands were visible on transverse postcontrast images, regardless of slice thickness. The LGs and TEGs were strongly contrast enhancing and were hyperattenuating compared to the zygomatic salivary gland. The LG was located dorsolaterally, with its anterior-most extent located under the orbital ligament, curving caudally to terminate caudal to the globe (Figure 1A). The TEG was oval in shape and conformed to the globe on the rostromedial aspect (Figure 1B). On precontrast images, the location of the glands could be identified by comparison to the postcontrast images, but were poorly defined. Conspicuity of the lacrimal glands was best on postcontrast thin-slice CT images in larger dogs. A 3D surface-rendering image was generated from 1.25 mm thickness, postcontrast CT images to demonstrate the relationships between the lacrimal glands, the globe, and the orbital ligament (Figure 2).

**Figure 3:**
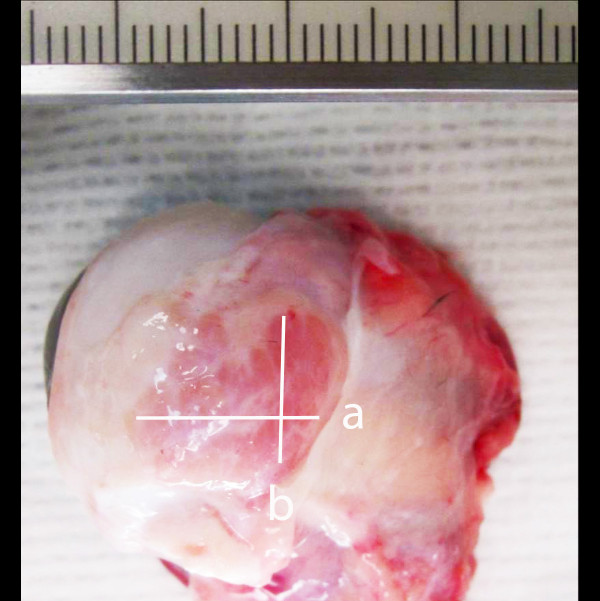
**Gross photograph of a specimen of the globe and surrounding tissues.** Cranial is to the left of the image. The lacrimal gland (white arrows) is visible as a thin, lobular structure in the dorsolateral tissues surrounding the globe. The length **(a)** and width **(b)** measurements of the lacrimal gland are indicated. Thickness was measured after dissection of the gland away from the globe (not shown).

In dogs with paired measurements of the LGs and TEGs, the measurements were found to be not statistically different between the left and right glands (P > 0.05). The measurements for volume, length, width, and height of the left and right LG and TEG were averaged and treated as a single measurement from each dog to remove within-animal correlations. The summary of measurements on imaging studies and gross specimens is presented in Table 1. The dissected specimens had similar measurements to those found in the imaging study, however width was overestimated on imaging, and length and height was underestimated (Figure 3).

**Table 1:** Summary of measurements of the canine accessory lacrimal gland of the third eyelid (TEG) and lacrimal gland (LG) on CT images

	**Glands**	**Mean**	**Std. Dev.**	**Min**	**Max**
**Volume (cm**^**3**^**)**					
TEG	41	0.10	0.05	0.30	0.22
LG	42	0.14	0.07	0.04	0.39
**Linear dimensions (mm)**					
TEG					
Height	41	2.02	0.60	1.16	3.84
Width	41	9.34	1.63	1.38	12.71
Length	41	7.90	2.52	4.00	13.75
LG					
Height					
Imaging	42	9.36	1.71	5.52	13.49
Gross specimen	6	10.48	1.69	8.05	13.75
Width					
Imaging	42	4.29	0.87	2.73	6.99
Gross specimen	6	2.32	0.68	1.42	3.54
Length					
Imaging	42	9.35	2.34	3.13	13.00
Gross specimen	6	11.61	1.98	7.52	13.38

The mean precontrast attenuation of the TEG was 46 HU (SD 10, 26–68) and the LG was 53 HU (SD 10, 26–69). The mean contrast enhancement on CT images of the TEG was 122 HU (SD 24, 59–179) and of the LG was 123 HU (SD 62, 62–159).

**Figure 4:**
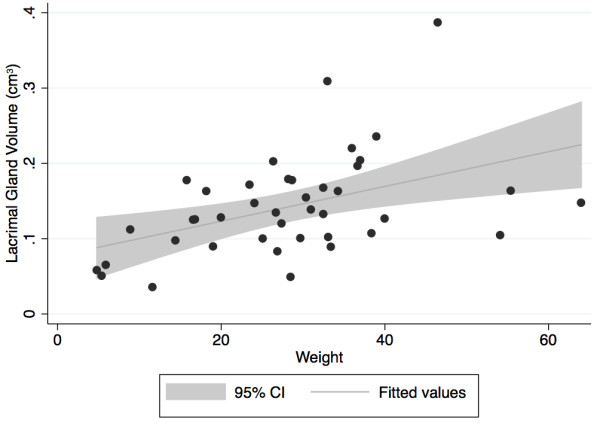
**Body weight and lacrimal gland volume are positively correlated [volume in cm**^**3**^ **= 0.0023 × (weight in kg) + 0.0771, R**^**2**^ **= 0.20, P = 0.0033].**

The regression model indicated that there was a significant positive correlation of dog weight and the volume measurement of the LGs [volume in cm^3^ = 0.0023 × (weight in kg) + 0.0771, R^2^ = 0.20, P = 0.0033] (Figure 4) and TEGs [volume in cm^3^ = 0.0021 × (weight in kg) + 0.0312, R^2^ = 0.28, P = 0.0008]. The slice thickness did not affect the volume measurement of the TEG or LG (R^2^ = 0.20, P = 0.50, P = 0.95).

## Discussion

The LGs and TEGs were well visualized on postcontrast CT images and appeared in consistent anatomical locations. The gland volumes increased with increasing body weight of the dog. The mean measurements of width, height, and length were described and were similar to measurements obtained from LGs in six cadavers.

Conspicuity of the lacrimal glands was best on thin-slice CT images in larger dogs. There was weaker correlation of body weight to LG volume in larger dogs, with both larger and smaller LG values than expected. This variability may need to be kept in mind when evaluating dogs with lacrimal gland disease, and further study could be considered in this group to investigate this trend. Although slice thickness did not have an effect on size measurement of the glands, the glands had similar imaging characteristics to soft tissue, and their small size prevented clear delineation from surrounding tissues on precontrast CT images. There were few brachycephalic dogs in the sample, however there were no apparent differences in the location or anatomy of the gland. The more rostral position of the globe within the orbit may cause slight alterations in the position of the gland relative to the globe. Rotation of the globe due to anesthesia should not affect the visibility of the glands, as the third eyelid remains relatively constant in position. The glands were static in their position relative to the osseous structures of the skull, and in the rostral to caudal direction relative to the globe.

The measurement of the TEG included the lymphoid tissue and cartilage of the third eyelid. These structures are intimately associated with the TEG and are contrast enhancing or hyperattenuating, and it was not possible to visually separate them. This should be taken into account when assessing TEG size on CT images. The left and right glands were symmetric within animals, as there were no differences in size detected. Asymmetry of the lacrimal glands could be an important indicator of unilateral disease if discrepancies are noted.

The mean LG width of the gross specimens was smaller than the mean width measured on imaging. The gross specimen population was uniform and comprised of beagle dogs weighing 11–12 kg. Since the size of the LG is positively correlated with body weight, the difference between the two populations may be due to the higher body weight of the clinical group. The mean length and height were smaller in the imaging group compared to the gross specimen. The edges of the gland thin progressively, and the peripheral portion of the gland may be below the resolution of the CT images, shortening height and length measurements.

The lacrimal glands (LG and TEG) are very small structures that have not been well studied using cross-sectional imaging in the dog. This characterization of their typical appearance will help to increase recognition of the glands and any associated abnormalities. Many ocular diseases, including neoplasia and inflammation, can extend to the structures within the orbit and may include the LG and/or the TEG. Recognition of gland involvement may help to alert clinicians to the possibility of dry eye. Diseases that have been reported involving the gland in people include pleomorphic adenoma, mucoepidermoid carcinoma, adenocarcinoma, epithelial cyst, dermoid cyst, lymphoid hyperplasia, autoimmune disease, acute dacryoadenitis, orbital cellulitis, and orbital pseudotumor [10]. Masses, including adenocarcinoma and lobular adenoma involving the lacrimal gland, have been reported in dogs [22,23].

Keratoconjunctivitis sicca is a common disease of the lacrimal apparatus in dogs, with etiologies including drug toxicity, distemper, multisystemic autoimmune disease, congenital disease, and idiopathic disease [5]. Assessment of the degree of tissue loss would ideally be performed through noninvasive imaging, as well as functional tests of tear production. With normal reference values available, diagnostic and serial imaging of patients becomes possible to monitor disease. In people, fatty replacement of the gland tissue occurs in Sjögren syndrome and has been visualized on MRI fat suppression sequences. Short T1 inversion recovery (STIR) sequences may have promise in evaluating the gland for fatty change using a prospective MR imaging protocol.

## Conclusions

In conclusion, the canine lacrimal glands are visible on postcontrast thin slice CT images, and normal reference measurements and volumes have been presented. Cross-sectional imaging may be a valuable tool in recognizing and evaluating lacrimal gland disease.

## Methods

CT images of dogs obtained from February 2010 to March 2011 were selected for measurement of the lacrimal glands. Images of dogs with no disease in the orbit or periorbital tissues within a 5 cm diameter were included. In this retrospective study, images of the head were obtained for clinically relevant reasons unrelated to the lacrimal gland when dogs were admitted to the Veterinary Medical Teaching Hospital. As such, ethical and owner approval was not required.

The transverse CT images were obtained pre- and postcontrast (Isovue 370 mg I/mL, Bracco diagnostics Inc., Bracco, NJ) on a multidetector row CT (Lightspeed 16, General Electric Co., Milwaukee, WI) in axial (20/42) or helical (22/42) mode. Images were acquired with a kV of 120 and a mean mA of 113 (±33) and were reconstructed in a soft-tissue algorithm. Image collimation ranged from 0.625-5 mm with a median of 2.5 mm without overlap or interslice gap.

The lacrimal glands were identified by their contrast enhancement relative to other orbital tissues and by their location in the rostromedial orbit (TEG) or caudolateral to the globe (LG). Linear and volumetric measurements of the glands were obtained on postcontrast images using image-viewing software (Osirix 3.8.1, Geneva, Switzerland). The TEG was measured using hand-drawn elliptical regions of interest for volume and linear measurements of width and height relative to the globe. The LG volume was measured with hand-drawn elliptical regions of interest, and width and height were measured with linear parallel and orthogonal measurements relative to the orbital ligament. Length measurements of the TEG and LG were calculated from the slice width multiplied by the number of slices in which the glands were visible to maximize spatial resolution on non-isotropic images. Maximum enhancement of the region of interest of each gland was recorded. Regions of interest were copied to the corresponding precontrast images to measure the attenuation of the poorly conspicuous TEG and LG. The mean CT attenuation was recorded with a region of interest in Hounsfield Units (HU).

To validate the size and location of the LGs, dissections were performed on six cadaver beagles weighing 11–12 kg. Measurements were not obtained for the TEGs. Use of the beagle dogs was approved by the Institutional Animal Care and Use Board of the University of California, Davis. These dogs were part of a larger study investigating the safety and immunomodulatory effects of mesenchymal stromal cells transplanted into the canine lacrimal gland, and euthanasia and necropsy was performed for this purpose [24].

Differences between measurements of the glands on the left and right sides were compared using the Wilcoxon signed-rank test with a P < 0.05 considered significant. A linear regression model was used to assess the effect of body weight, slice thickness, and modality on volume measurements of the LGs and TEGs. Analysis was performed using commercial software (StataCorp. 2009. *Stata Statistical Software: Release 11*. College Station, TX: StataCorp LP).

## Abbreviations

LG: Lacrimal gland; TEG: Accessory lacrimal gland of the third eyelid; CT: Computed tomography.

## Competing interests

The authors declare that they have no competing interests.

## Authors’ contributions

All authors (ALZ, SP, CJM) participated in the design and conception of the study, drafting and revision of the manuscript, gave final approval of the version to be published, and agreed to be accountable for all aspects of the work. ALZ and SP performed the collection and interpretation of data.
